# Metrological framework for selecting morphological characters to identify species and estimate developmental maturity of forensically significant insect specimens

**DOI:** 10.1080/20961790.2020.1794347

**Published:** 2020-09-10

**Authors:** John Mark Midgley, Martin Herrer Villet

**Affiliations:** a Southern African Forensic Entomology Research Laboratory, Department of Zoology and Entomology, Rhodes University, Makhanda, South Africa; b Department of Natural Sciences, KwaZulu-Natal Museum, Pietermaritzburg, South Africa

**Keywords:** Forensic sciences, forensic entomology, identification, individuation, data types, metrology, measurement error, repeatability

## Abstract

Accurate age estimates of immature necrophagous insects associated with a human or animal body can provide evidence of how long the body has been dead. These estimates are based on species-specific details of the insects’ aging processes, and therefore require accurate species identification and developmental stage estimation. Many professionals who produce or use identified organisms as forensic evidence have little training in taxonomy or metrology, and appreciate the availability of formalized principles and standards for biological identification. Taxonomic identifications are usually most readily and economically made using categorical and qualitative morphological characters, but it may be necessary to use less convenient and potentially more ambiguous characters that are continuous and quantitative if two candidate species are closely related, or if identifying developmental stages within a species. Characters should be selected by criteria such as taxonomic specificity and metrological *repeatability* and *relative error*. We propose such a hierarchical framework, critique various measurements of immature insects, and suggest some standard approaches to determine the reliability of organismal identifications and measurements in estimating postmortem intervals. Relevant criteria for good characters include high repeatability (including low scope for ambiguity or parallax effects), pronounced discreteness, and small relative error in measurements. These same principles apply to individuation of unique objects in general.

**Key points**
- Metrological rigour can increase in forensic entomology by selecting measurements based on their metrological qualities.
- Selection of high-quality features for morphological identification of organisms should consider these criteria: (1) pronounced discreteness of features (minimising group overlap or maximizing interval); (2) high repeatability of assessment (such as symmetrical width rather than asymmetrical length); (3) small relative error in measurement (selecting the physically largest continuous rigid feature for measurement).
- These metrological principles also apply to individuation of unique objects in general.

## Introduction

Immature insects associated with a corpse can provide evidence of the time since death if their ages can be inferred based on their species and developmental stage [1–5]. However, many forensic professionals who generate such forensic evidence and most legal professionals who cite these identifications and estimates have little explicit training in taxonomy or metrology. In our experience, they appreciate having access to formalized principles and standards for biological identification. The call for such standards is also a hallmark of quality management in these professions [6, 7]. This review addresses these needs.

Useful studies exist on the identification and development of eggs [8–10], larvae [11–19], and pupae [3, 20, 21]. Although estimating adults’ ages is possible [22–24], it has minimal practical applicability. Identification and age estimation can be done at the expense of time and money using molecular characters such as DNA [25] or RNA [20] sequences, Matrix-Assisted Laser-Desorption and Ionization Time-of-Flight mass spectrometry [26], or possibly cuticular hydrocarbons [27]; alternatively, it can be done more cheaply using morphological characteristics (usually termed *character states* in taxonomy).

**Figure 1.**
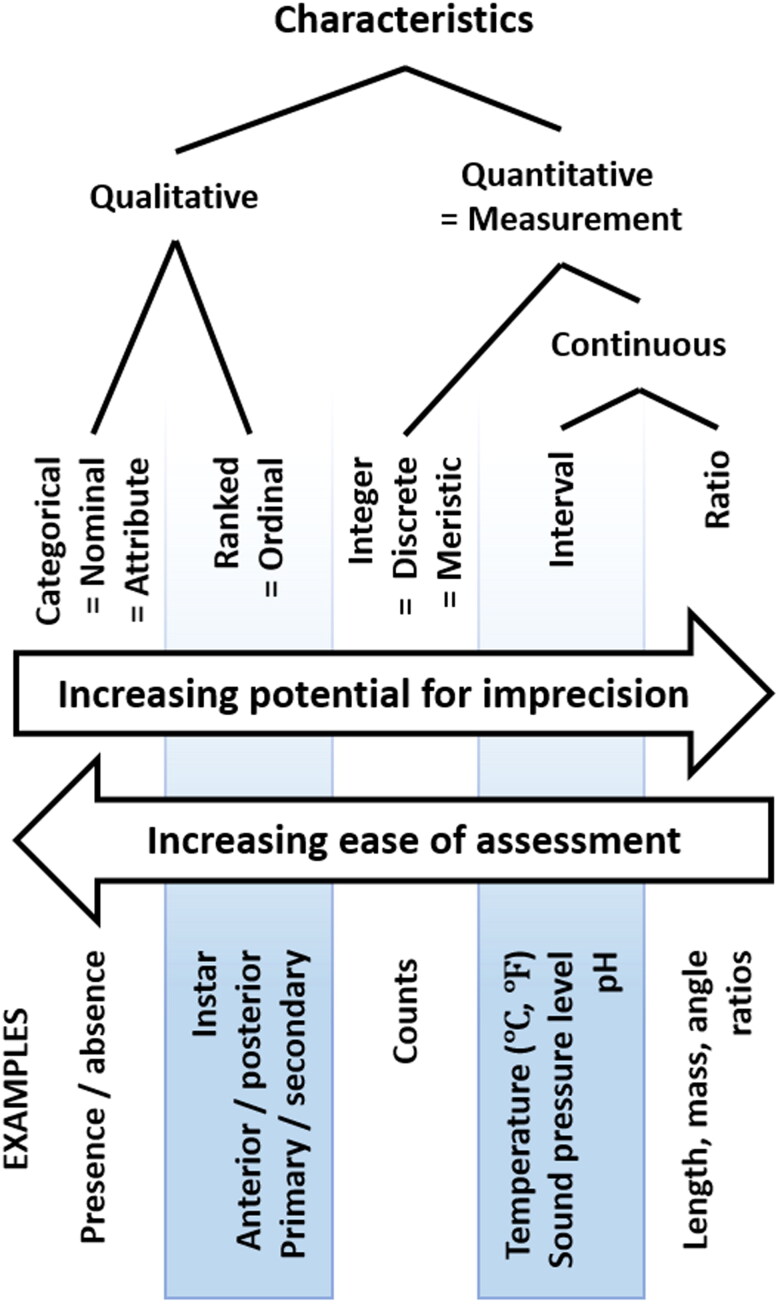
Metrological framework for classifying traits used to identify insects and estimate the ages of their immature stages.

Metrology is the science of making reliable measurements, and is relevant to the use of morphological characters in forensic entomology to produce evidence that meets the quality standards of the courtroom [28]. Metrologically, morphological characters can be qualitative or quantitative, and discrete or continuous (Figure 1, Table 1).

**Table 1.** Typology and examples of characters for determining species and instars.

Data type	Examples	Source
Qualitative		
Categorical	Presence of seta, spinose bands broken or complete	Szpila and Villet 2011 [19]
	Sternite shape, urogomphus shape, mouthpart shape	Daniel et al. 2017 [11]
Ranked	Eye colour, setae colour, antennal colour	Brown et al. 2015 [3]
		
Quantitative		
Meristic	Number of setae on spiracle and on mandible	Daniel et al. 2017 [11]
	Number of teeth on mouthhook	Szpila and Villet 2011 [19]
Measurements	Pronotum width, mesonotum width, distance between dorsal stemmata	Frątczak and Matuszewski 2014 [29]
	Body length, body width	Ridgeway et al. 2014 [12]
	Head capsule width, head capsule length	Daniel et al. 2017 [11]
	Urogomphus segment length ratio, urogomphus:tergite 10 length ratio	Daniel et al. 2017 [11]

*Categorical* (or *nominal* or *attribute*) traits are qualitative, discrete, and lack both magnitude and rational order or sequence [30], e.g. the presence or absence of features, and are generally easy to decide. In systematics, they are often binary.

*Ranked* (or *ordinal*) traits are qualitative and discrete like categorical traits but have relative magnitude and can therefore be placed in a sequence [30], e.g. anterior or posterior positions. They lack absolute magnitude, and therefore cannot be measured. They are usually easy to decide if observations are not close to being tied (as horse racing enthusiasts will attest).

*Quantitative* (or *measurement*) characters have absolute magnitude, usually expressed in standard units, and may be *discrete* (or *meristic*: counted in integers) or *continuous* (measured in real numbers) [30]. Some continuous quantitative traits (e.g. colour measured as light wavelengths) may be functionally discrete or even categorical (e.g. colour due to the presence or absence of causative pigment). Continuous traits can be measured on *interval* scales that have a relative baseline and negative values (e.g. the Celsius, Fahrenheit, pH, and decibel scales) or on *ratio* scales with an absolute baseline, where a value of zero means that there is nothing to measure (e.g. the Kelvin scale). Only ratio scales can produce arithmetically meaningful ratios [31].

Continuous variables may show variation for several reasons, one of which is imprecision (also technically termed *error*) in the measuring process, which is typically addressed using statistical analysis. Statistical analyses of ratios and angles need to be conducted with particular attention, because ratios, proportions, and angles can have non-Gaussian distributions that do not meet the assumptions for some analyses. Qualitative traits are thus easier to assess than continuous quantitative traits (Figure 1).

Two concepts are germane to assessing imprecision. *Repeatability* refers to whether multiple independent measurements of one observation will produce the same result (as opposed to individual measurement of multiple observations, which are termed replicates). *Relative error* refers to what proportion of a measurement is attributable to the resolution limit of the measuring device. If the resolution limit of a measuring device is fixed (e.g. at 1 mm), its relative error is smaller for large measurements (e.g. 1% for 100 mm) than for smaller measurements (e.g. 10% for 10 mm). Both of these concepts affect the choice of diagnostic traits for forensic identification and estimation.

Qualitative features (such as mouthpart shape [11] or colouration [32]) might be subtly different between some related species and clearly different between others. However, typically, they do not drastically differ between instars within a species, which makes quantitative measures of maturity necessary. The shape or colour of a feature can be quantitatively described by morphometrics [33, 34] or spectrometry, respectively. Pupal development does not progress through discrete stages, but the development of certain features (such as seta formation or setal pigmentation) can be treated as discrete developmental landmarks at sufficiently coarse time scales and continuous developmental processes at finer temporal resolutions [3].

Ideal identifying characters are unambiguous and categorical, such as the presence of setae or absence of spinose bands on the cuticle [18, 19], or meristic (quantitative and discrete), such as the number of slits or buttons in the posterior spiracles [35] or the number of teeth on the mouth hooks [19]; all of these are used as diagnostic traits of blow fly larvae (Table 1).

Although character selection in modern taxonomic research is generally robust, these studies typically address the adult stages of sister taxa [36, 37]. In contrast, the early descriptions on which modern taxonomy is built are regularly not particularly informative because the authors could not know which traits would become significant in the future. Similarly, many forensic studies that focused on larvae effectively used circular logic by choosing features based on the significance to their own results without assessing the metrological characteristics of the traits [29, 38]. Multiple features are often tested, with the most significant retained and the source of unreliability in other measures not investigated. However, the measure could be inherently poor (e.g. poor repeatability) or poorly applied (e.g. relative error).

The implications of these ideas is illustrated by a case study of this metrological framework to assess the use of different types of morphological characters to differentiate between species and between conspecific instars of carrion beetles of the genus *Thanatophilus* Leach. We then suggest a general strategy for the selection of identifying characters that is applicable in forensic identification. This strategy can be extended to non-entomological objects that require physical characterization, e.g. for forensic individuation.

## A case study: *Thanatophilus* beetles

### Differentiating species

The adults of at least 19 species can be identified by combinations of qualitative characteristics of colouration, the presence or absence of tubercles, and the shapes of the male genitalia, female propygidia, and the sexually dimorphic forewing apices [39]. Using Schawaller’s [39] study, shape characters can be assessed without specialized equipment or measurement, let alone skill in deploying them, by making comparisons with diagrams that Schawaller specifically laid out to facilitate them.

**Figure 2.**
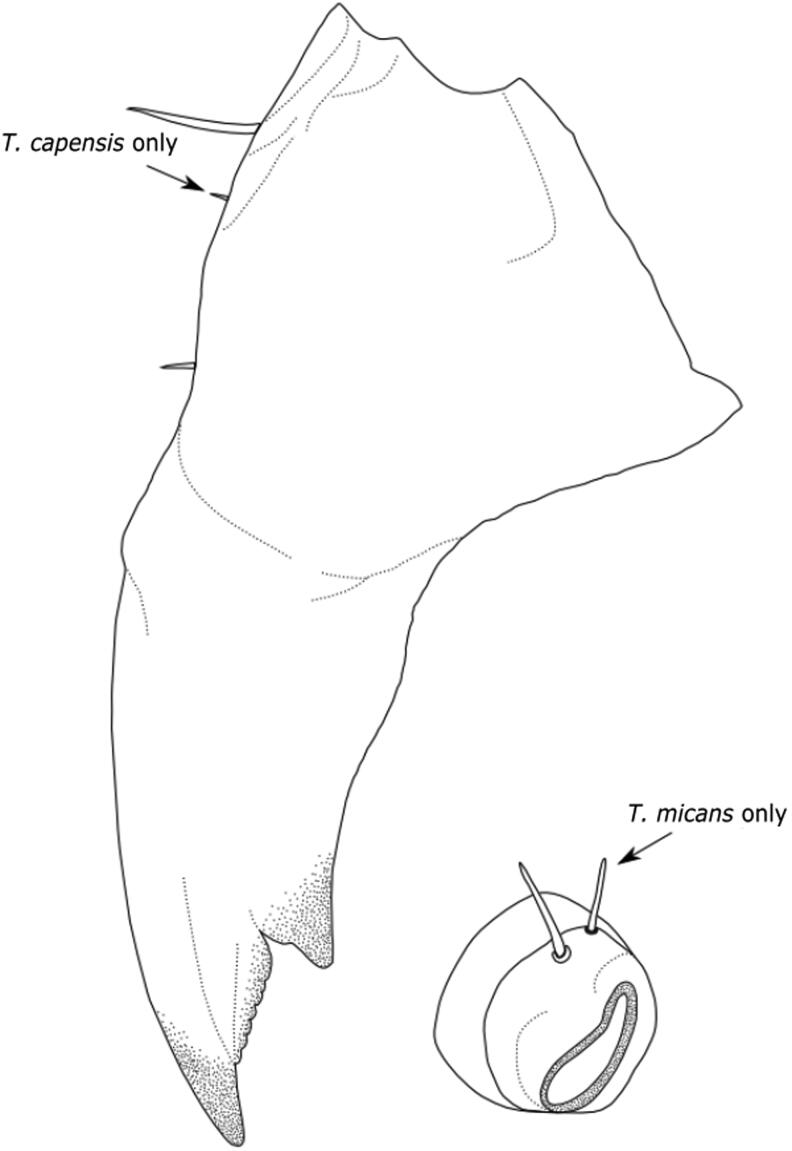
Key features of *Thanatophilus* larvae. Mandible of *T. capensis*, showing three setae (two in *T. micans*), and mesothoracic spiracle of *T. micans*, showing two setae (one in *T. capensis*). Image from Daniel et al. 2017 [11] under Creative Commons Attribution Licence (CC BY 4.0).

The known larvae of *Thanatophilus* have fewer known qualitative morphological characters [11, 14, 17, 32, 40, 41], and those characters which have been identified are currently only sufficient for identifying the larvae of closely related *Thanatophilus* species (Figure 2, Table 2). This provides a potentially interesting model to explore continuous quantitative characters that might be useful for identifying species and instar, using morphometrics.

**Table 2.** Characters for identifying *Thanatophilus* larvae.

Character	*T. capensis* [11]	*T. micans* [11]	*T. sinuatus* [17]	*T. rugosus* [17]	*T. trituberculatus* [41]	*T. lapponicus* [32, 41]	*T. coloradensis* [32]
Qualitative							
1st instar palpi			Shorter	Longer			
2nd instar prothoracic paratergite spot			Translucent	Brown			
3rd instar paratergites	Dark	Dark	White	Dark		Dark	Yellowish
abdominal sternite 5 anterior margin	Straight	Convex					
Meristic							
Mandibular setae	3	2	2	2			
Mesothoracic spiracular setae	1	2	1	1			
Ratio							
Urogomphus length : abdominal segment 10 length					2.5 – 3.0	<2.0	<2.0
Urogomphus length 1st : 2nd segments			∼2.3	∼2.2	∼1.5	2.0 – 3.0	2.0 – 3.0

Accounts of development in *Thanatophilus* have focused on categorical developmental events rather than on continuous, quantitative indicators of maturity [12–14, 17]. In captivity, it is easy to determine which instar a larva has reached, because the advent of ecdysis (or larval moulting) is marked by the appearance of an exuvium (the moulted exoskeleton) in the rearing chamber [12]. However, the instars of larvae sampled directly from a corpse can be harder to differentiate, and continuous, quantitative measurements of growth are often required. Because *Thanatophilus* larvae can be reared individually [12], they produce more accurate experimental data than flies, which must be reared communally [42]; communal rearing does not allow the same individual to be repeatedly measured, or the identification of sick specimens [12].

## Materials and methods

To differentiate the species and larval instars, specimens of each instar of *Thanatophilus micans* and *T. capensis* (= *T. mutilatus*) were taken from laboratory colonies held at the Department of Zoology and Entomology, Rhodes University and drowned in ethanol, which is the preferred method for preserving forensic samples of sclerotized beetle larvae [43]. Species identification information was taken from Daniel et al. [11]. Additional heads and urogomphi (posterior horns) were mounted in Euparal on microscope slides to augment the data from Daniel et al. [11]. Head capsule width and urogomphus length of at least 11 specimens of each species–instar combination were measured using a Wild M5A stereomicroscope (Wild Heerbrugg, Switzerland) with a reticle. Body length and width measurements were taken from Ridgeway et al. [12], and were measured with a simple gauge [44, 45] precise to 0.1 mm. General size and shape observations were made while collecting data for experiments in both Midgley and Villet [13] and Ridgeway et al. [12].

## Results

Larvae increased in length, width, and volume as they grew, and changed shape. Early in an instar, their body cross-section was relatively flattened, but it became more circular as their digestive tracts filled. Cessation of feeding prior to ecdysis resulted in a slight flattening, and sometimes also a telescopic shortening, of the body. Immediately after ecdysis, the body became wider as the exoskeleton became larger. This resulted in a further shortening and flattening of body length to maintain total body volume. To achieve an approximately constant volume despite widening of the body, the abdomen telescoped into itself, which made the abdominal taper sharper and steeper stepped. The length of the abdomen compared with the thorax allometrically increased as the larvae grew, presumably because the fat bodies enlarged.

Body length rapidly increased once feeding resumed. The change to a rounder body profile happened more slowly and was most noticeable closer to the following ecdysis. In mature final instar larvae, the body was most noticeably rounded and elongate as the body stretched to prepare for pupation. As the intersegmental membranes became stretched, the abdomen appeared more gradually and evenly tapered.

**Figure 3.**
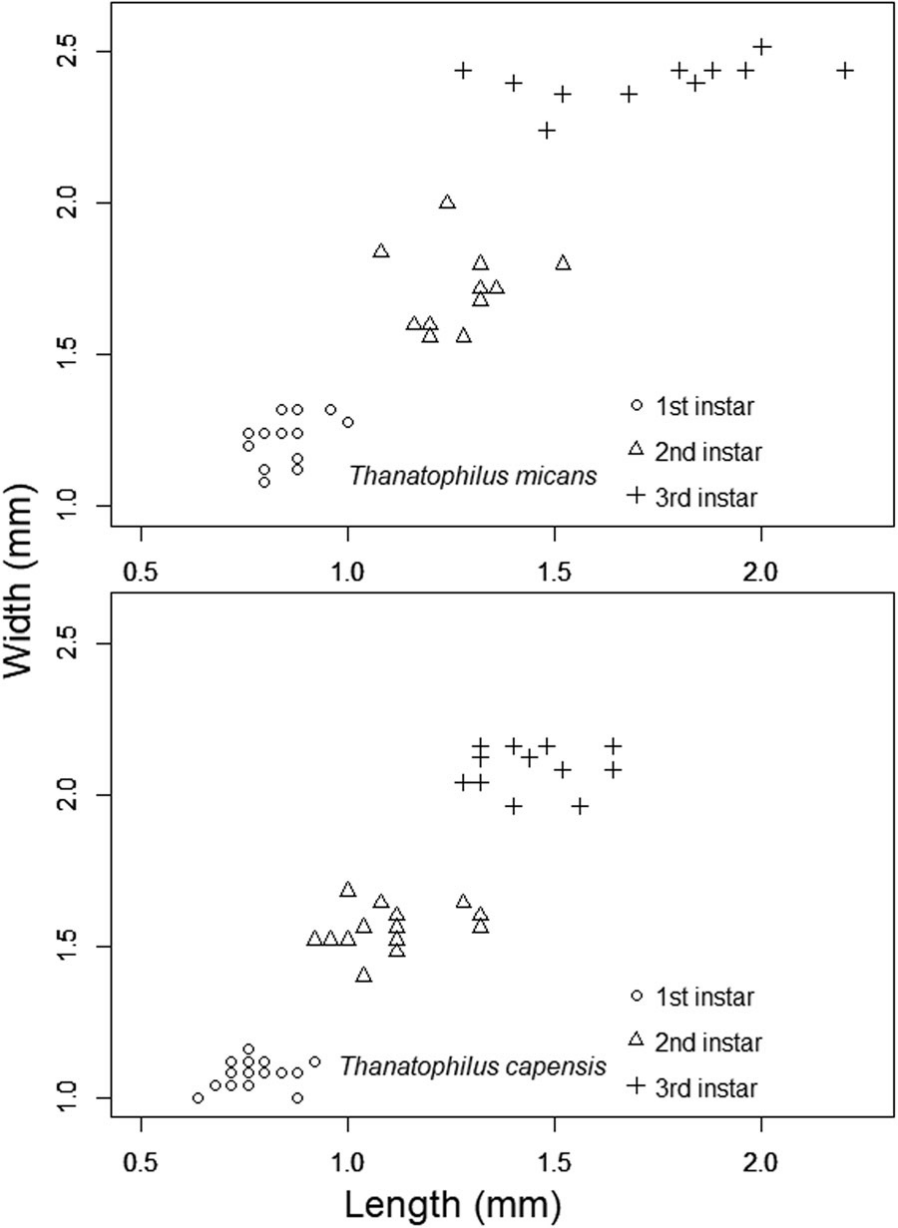
Head capsule width and length in *Thanatophilus micans* and *T. capensis* larvae. Length shows greater variation than width because of lower measurement repeatability. Width and length show similar arrangement into rows or columns because their relative errors are similar (approximately 5%). Image from Daniel et al. 2017 [11] under Creative Commons Attribution Licence (CC BY 4.0).

Qualitative characteristics that differentiate the instars of Afrotropical *Thanatophilus* species were not noted [11], and instar was most reliably quantitatively determined from head capsule width. In contrast, head capsule length significantly overlapped between instars and was erratic within instars (Figure 3). This was because it was difficult to orientate the capsule in a standard position because it tipped up to variable degrees; this led to parallax effects [46] and unreliability of this particular measurement, because there was low repeatability.

**Figure 4.**
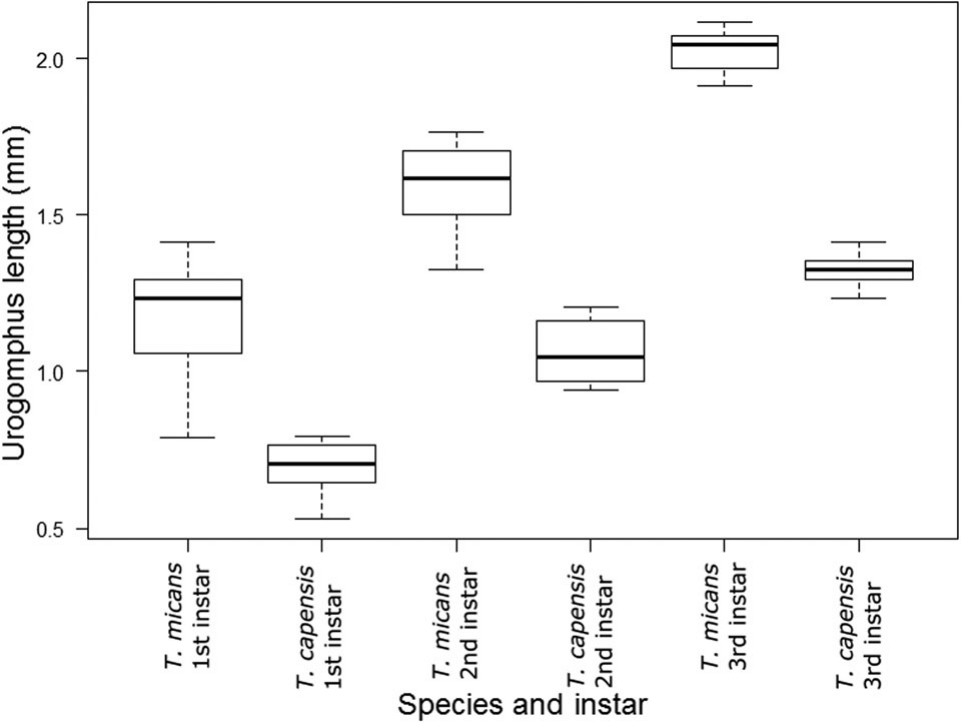
Urogomphus length in *Thanatophilus micans* and *T. capensis* larvae. Although first instar larvae show slight overlap between species, second and third instar larvae show discrete grouping.

**Figure 5.**
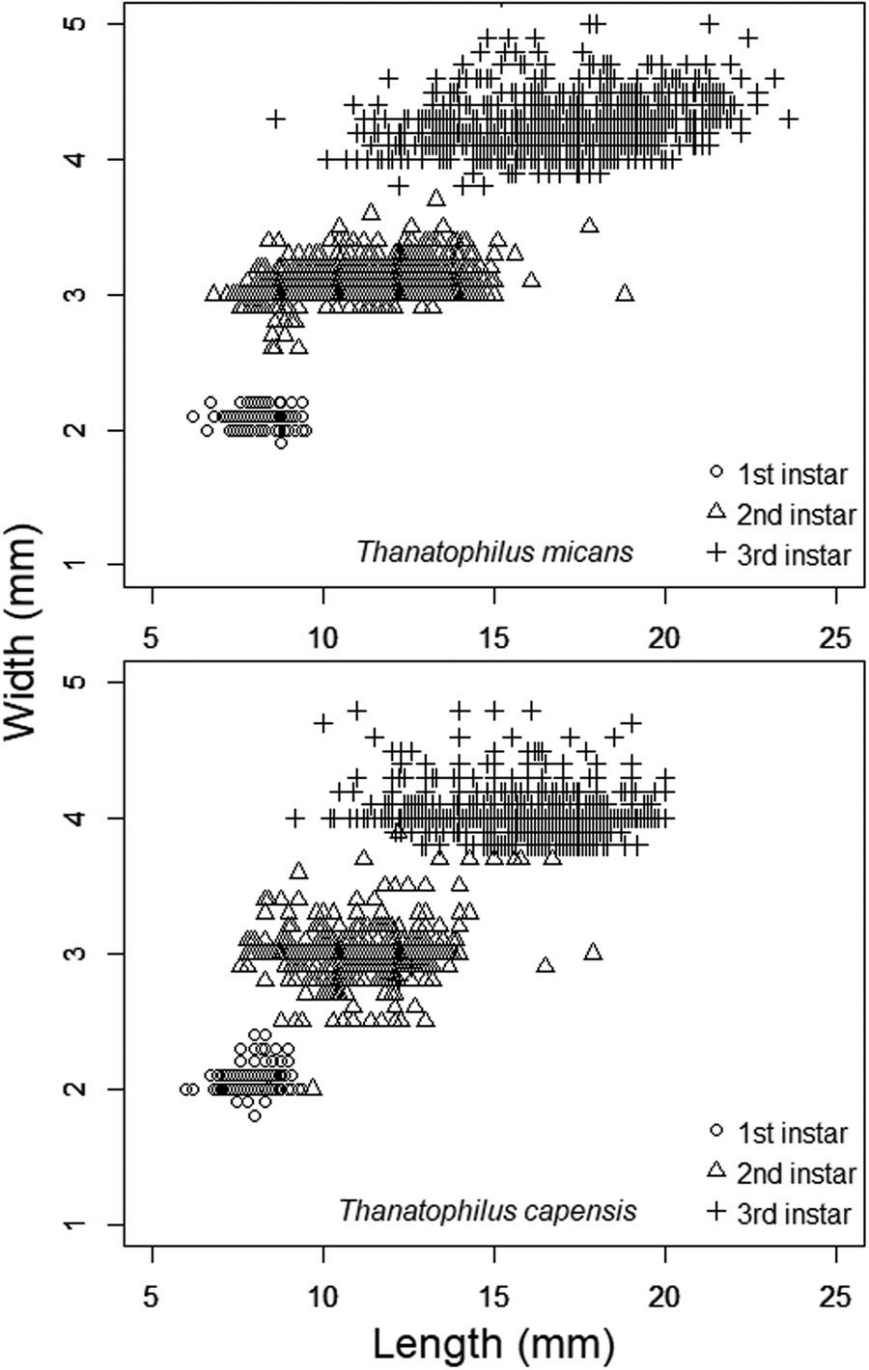
Body length and width in *Thanatophilus micans* and *T. capensis* larvae. Although width shows discrete grouping by instar, length significantly overlaps. Length and width both overlap between species. Width shows obvious groups, whereas length does not, because its relative error is approximately four times smaller.

Maximum urogomphus length (Figure 4) or thoracic width (Figure 5) were more repeatable and moderately discriminatory; however, in several cases, these traits showed overlap between instars and could not be reliably used. The absolute range of measurement for body width was consistent between instars, which indicates that the method shows repeatability.

Total body length showed significant overlap between instars in both species (Figure 5). Because the body is extensively jointed between segments, it showed subtle postural distortions, even if the larvae were killed in a manner that minimized distortion and standardized posture as much as possible [43].

Inevitably, if body length and width are measured with the same instrument at the same magnification, the relative error in the larger measurement will be smaller. Body length is thus effectively measured at a coarse scale of resolution, which gives it the appearance of being less variable [47]. This effect is shown in Figure 5, where the width, with high relative error, appeared to have a quantized distribution and formed rows in the plot. The smaller relative error in length gave the distribution a more even resolution and did not show distinct columns.

## Discussion

Recent publications on forensically important insect larvae have addressed aspects of larval preservation and measurement [29, 43, 48, 49]. However, standard measurements or a metrological framework for standard criteria to select features for such measurement have not been established, despite recent concern [50]. It is often assumed that measurements should be continuous–quantitative because growth is a continuous process. However, meristic and qualitative measurements are also relevant to identification. In some cases, continuous–quantitative measures can even be undesirable because the metrological precision exceeds the biological meaningfulness of the measure. This can be exacerbated by averaging of multiple samples, which creates false arithmetic precision, such as in developmental models based on communally-reared maggots.

Quantitative measures are also not independent of allometric variation, and measurement of rigid structures that are metrically stable between moults is preferable for instar determination. Usually, qualitative traits are useful for determining species, whereas continuous–quantitative measurements are more useful for determining instar. Meristic traits can be useful for both because some features increase in number with growth, and differences between species can also be incremental. Thoughtful selection of measurable characters is needed to sufficiently and reliably determine both species and instar for presentation in court. Relevant criteria for good measurements include high repeatability, pronounced discreteness, and small relative measuring error. This also applies to individuation of single objects.

### Natural history of size and shape of larvae

Our observations on *Thanatophilus* larvae size and shape are the most detailed about beetles to date in the forensic entomology literature. Observations of immature insects in published studies were usually superficial [14, 17, 51, 52], and these changes in size and shape were sometimes not even discussed, despite being visible in figures [53]. Additionally, average measurements at a given time point or long measurement intervals can disguise changes in shape or the magnitude of these changes. Because beetle larvae are kept in solitary conditions, repeated measures of length showed changes in shape more clearly (see Figure 2 in Ridgeway et al. [12]).

### Species determination

The larvae of Afrotropical *Thanatophilus* species can be easily separated using categorical (sternite and urogomphus shape), meristic (thoracic setae), or continuous–quantitative (ratio of urogomphus length:sternite 10 length) features [11]. Although the length of the urogomphus showed overlap between instar–species combinations (Figure 4), using a ratio to assess this character reduced the effect of allometric variation to an acceptable level [11, 14, 17]. Characters used in species determination are usually limited to categorical or meristic features, but it is actually the discreteness of a feature that is important. In Afrotropical *Thanatophilus*, urogomphus length shows continuous intraspecific variation, but discrete groups for each species, which makes it a useful measure for species determination. Multiple types of characters were also used in Rognes and Paterson’s [36] unusually detailed treatment of *Chrysomya chloropyga* and *C. putoria*. In this case, it was meant to settle a 50-year-old disagreement over the status of *C. putoria*; however, traditionally, the use of continuous measures in taxonomy has been limited. Rognes and Paterson [36] even expressed “surprise” (p. 56) at the usefulness of this feature type.

### Instar determination

As in previous studies on larvae of European *Thanatophilus* species [40], the three measures used in this study showed different utility in determining instar. Measurement repeatability was illustrated by the head dimensions. Measuring head capsule width is easily repeated because misaligned specimens appear asymmetrical, which make it easy to achieve standardized orientation and measurement. Head capsule length is more difficult to measure because symmetry cannot help to assess whether a specimen is correctly orientated, which makes standardized, repeatable measurements less likely because of parallax errors. In this study, this was exemplified by the larger variation found within instars, and the larger degree of overlap between instars in head length compared with width (Figure 3). This disparity in repeatability also has relevance for morphometrics because assessment of head capsule shape is only as repeatable as the least repeatable measure, in this case the head capsule length. The parallax effect has been shown to be significant in morphometrics [46, 54]. Additionally, the same problem has been recognized in proturan taxonomy [55]. The concern of repeatability is not limited to continuous variables. Williams and Villet [37] identified the angle (right or obtuse) formed by the vertical and prevertical setae as a useful categorical feature; however, the angle can be difficult to determine if the specimen is not optimally orientated.

Urogomphus length and thoracic width are less reliable measures. In most cases, instar determination is possible using these measures, but some overlap does occur. Thoracic width overlapped less often than urogomphus length, but neither of these measures could be classified as discrete. Thoracic width is more repeatable than urogomphus length and head capsule length because of orientation symmetry.

Unlike the dimensions of rigid exoskeletal structures (like the head) that showed discrete growth, body length changed continuously as larvae grew and showed notable overlap between instars in both species in this study (Figure 5). Part of this overlap is due to the small loss of body size just prior to ecdysis [12], which is while the larva is not feeding and its gut empties, and also after ecdysis, when the body is wider but has the same total volume. These overlaps are not attributable to measurement error. Additional error was introduced by postural distortion. Although these are measurement errors, they are currently unavoidable because the recommended best practice was used [43]. These effects completely overwhelmed the improvement in relative error that is offered by using the much larger size of the body rather than either thorax or head width. For these reasons, this type of dimension is not recommended for assessing instar or age.

Finally, if the precision limit of a measuring instrument is constant, e.g. to the nearest 0.1 mm, the *relative* error of a measurement will be smaller for larger measurements, as has been noted in fisheries research [56]. It is important to either use a more precise measuring tool for small features, such as a microscope with an ocular micrometre over a measuring gauge; alternatively, if one is not available, care should be used when interpreting overlap in measurement. A given measuring technique is likely to have a higher relative error when measuring younger and smaller individuals, as illustrated in Figure 4. The absolute range of the body width measurements was consistent, despite older instars being larger, which indicates higher relative error in smaller measurements.

## Conclusion

The selection of features for measurement depends on the aim of the measurement. Measures that proved useful for separating *Thanatophilus* species were all qualitative and had no use in determining instar, which was most effectively identified using quantitative measures of the rigid parts of the exoskeleton. Quantitative measurement of the telescopic body parts was least useful. It is also not possible to identify a single feature that should be measured for all forensically important taxa. In fact, it is not even possible to suggest a preference for qualitative or quantitative features. Instead, our data show that high-quality feature selection should focus on the following selection criteria:

1. Pronounced discreteness (minimizing overlap or maximizing interval);

2. High repeatability (such as symmetrical width rather than asymmetrical length);

3. Small relative error (selecting the physically largest continuous rigid feature for measurement).

By selecting measurements based on their quality, rather than the resulting data type, metrological rigour will be increased in forensic entomology. These same metrological principles apply to individuation of unique objects in general.

## References

[1] Villet MH, Richards CS, Midgley JM. Contemporary precision, bias and accuracy of minimum post-mortem intervals estimated using development of carrion-feeding insects. In: Amendt J, Goff M, Campobasso C, editors. Current concepts in forensic entomology. Dordrecht (the Netherlands): Springer Netherlands; 2010. p. 109–137.

[2] Villet MH, Amendt J. Advances in entomological methods for death time estimation. In: Turk EE, editor. Forensic Pathology Reviews, Vol 6. Heidelberg (Germany): Humana Press; 2011. p. 213–238.

[3] Brown K, Thorne A, Harvey M. *Calliphora vicina* (Diptera: Calliphoridae) pupae: a timeline of external morphological development and a new age and PMI estimation tool. Int J Legal Med. 2015;129:835–850. 25209716 10.1007/s00414-014-1068-z

[4] Rozane B, Martin HV. The uses of *Chrysomya megacephala* (Fabricius, 1794) (Diptera: Calliphoridae) in forensic entomology. Forensic Sci Res. 2018 Mar 21;3:2–15. 30483647 10.1080/20961790.2018.1426136 PMC6197084

[5] Lipin R, Yanjie S, Wei C, et al. A brief review of forensically important flesh flies (Diptera: Sarcophagidae). Forensic Sci Res. 2018 Mar 22;3:16–26. 30483648 10.1080/20961790.2018.1432099 PMC6197121

[6] Committee on Identifying the Needs of the Forensic Sciences Community NRC. Strengthening forensic science in the United States: a path forward. Washington, DC: The National Academies Press; 2009.

[7] Amendt J, Anderson G, Campobasso CP, et al. Standard practices. In: Tomberlin J, Benbow M, editors. Forensic Entomol Int Dimens Front. Boca Raton (FL): CRC Press; 2015. p. 381–398.

[8] Sukontason K, Sukontason KL, Piangjai S, et al. Identification of forensically important fly eggs using a potassium permanganate staining technique. Micron. 2004;35:391–395. 15006363 10.1016/j.micron.2003.12.004

[9] Martín-Vega D, Hall M. Estimating the age of *Calliphora vicina* eggs (Diptera: Calliphoridae): determination of embryonic morphological landmarks and preservation of egg samples. Int J Legal Med. 2016;130:845–854. 26753872 10.1007/s00414-015-1308-x PMC4830879

[10] Meskin I. Factors affecting the coexistence of blowflies (Diptera: Calliphoridae) on the Transvaal Highveld. South Afr J Sci. 1986;82:244–250.

[11] Daniel CA, Midgley JM, Villet MH. Determination of species and instars of the larvae of the Afrotropical species of *Thanatophilus* Leach, 1817 (Coleoptera, Silphidae). African Invertebrates. 2017;58:1–10.

[12] Ridgeway JA, Midgley JM, Collett IJ, et al. Advantages of using development models of the carrion beetles *Thanatophilus micans* (Fabricius) and *T. mutilatus* (Castelneau) (Coleoptera: Silphidae) for estimating minimum post mortem intervals, verified with case data. Int J Legal Med. 2014;128:207–220. 23974525 10.1007/s00414-013-0865-0

[13] Midgley JM, Villet MH. Development of *Thanatophilus micans* (Fabricius 1794) (Coleoptera: Silphidae) at constant temperatures. Int J Legal Med. 2009;123:285–292. 18779975 10.1007/s00414-008-0280-0

[14] Novák M, Jakubec P, Qubaiová J, et al. Revisited larval morphology of *Thanatophilus rugosus* (Coleoptera: Silphidae). Int J Legal Med. 2018;132:939–954. 29270839 10.1007/s00414-017-1764-6

[15] Villet MH, MacKenzie B, Muller WJ. Larval development of the carrion-breeding flesh fly *Sarcophaga* (*Liosarcophaga*) *tibialis* Macquart (Diptera: Sarcophagidae) at constant temperatures. African Entomol. 2006;14:357–366.

[16] Lefebvre F, Pasquerault T. Temperature-dependent development of *Ophyra aenescens* (Wiedemann, 1830) and *Ophyra capensis* (Wiedemann, 1818) (Diptera, Muscidae). Forensic Sci Int. 2004;139:75–79. 14687777 10.1016/j.forsciint.2003.10.014

[17] Jakubec P, Novák M, Qubaiová J, et al. Description of immature stages of *Thanatophilus sinuatus* (Coleoptera: Silphidae). Int J Legal Med. 2019; 133:1549–1565. 30879134 10.1007/s00414-019-02040-1

[18] Szpila K, Richet R, Pape T. Third instar larvae of flesh flies (Diptera: Sarcophagidae) of forensic importance—critical review of characters and key for European species. Parasitol Res. 2015;114:2279–2289. 25823900 10.1007/s00436-015-4421-3 PMC4430590

[19] Szpila K, Villet MH. Morphology and identification of first instars of African blow flies (Diptera: Calliphoridae) commonly of forensic importance. J Med Entomol. 2011;48:738–752. 21845931 10.1603/me10238

[20] Boehme P, Spahn P, Amendt J, et al. Differential gene expression during metamorphosis: a promising approach for age estimation of forensically important *Calliphora vicina* pupae (Diptera: Calliphoridae). Int J Legal Med. 2013;127:243–249. 22555870 10.1007/s00414-012-0699-1

[21] Brown K, Harvey M. Optical coherence tomography: age estimation of *Calliphora vicina* pupae *in vivo*? Forensic Sci Int. 2014;242:157–161. 25064575 10.1016/j.forsciint.2014.07.001

[22] Butcher JB, Moore HE, Day CR, et al. Artificial neural network analysis of hydrocarbon profiles for the ageing of *Lucilia sericata* for post mortem interval estimation. Forensic Sci Int. 2013;232:25–31. 24053861 10.1016/j.forsciint.2013.06.018

[23] Moore H, Adam C, Drijfhout F. Potential use of hydrocarbons for aging *Lucilia sericata* blowfly larvae to establish the postmortem interval. J Forensic Sci. 2012;58:404–412. 23130882 10.1111/1556-4029.12016

[24] Hayes EJ, Wall R, Smith KE. Measurement of age and population age structure in the blowfly, *Lucilia sericata* (Meigen) (Diptera: Calliphoridae). J Insect Physiol. 1998;44:895–901. 12770425 10.1016/s0022-1910(98)00067-5

[25] Tarone A, Singh B, Picard C. Molecular biology in forensic entomology. In: Tomberlin J, Benbow M, editors. Forensic entomology international dimensions and frontiers. Boca Raton (FL): CRC Press; 2015. p. 297–316.

[26] Reeve MA, Buddie AG, Pollard KM, et al. A highly-simplified and inexpensive MALDI-TOF mass spectrometry sample-preparation method with broad applicability to microorganisms, plants, and insects. J Biol Methods. 2018;5:103. 10.14440/jbm.2018.261 PMC6706156 31453253

[27] Moore HE, Butcher JB, Day CR, et al. Adult fly age estimations using cuticular hydrocarbons and Artificial Neural Networks in forensically important Calliphoridae species. Forensic Sci Int. 2017;280:233–244. 29107219 10.1016/j.forsciint.2017.10.001

[28] Lentini JJ. Forensic science standards: where they come from and how they are used. Forensic Sci Policy Manag An Int J. 2009;1:10–16.

[29] Frątczak K, Matuszewski S. Instar determination in forensically useful beetles *Necrodes littoralis* (Silphidae) and *Creophilus maxillosus* (Staphylinidae). Forensic Sci Int. 2014;241:20–26. 24835031 10.1016/j.forsciint.2014.04.026

[30] Sokal RR, Rohlf FJ. Introduction to biostatistics. 2nd ed. Mineola (NY): Dover Publications; 2009.

[31] Saaty TL. What is relative measurement? Math Comput Model. 1993;17:1–12.

[32] Anderson RS, Peck SB. The carrion beetles of Canada and Alaska. Coleoptera: Silphidae and Agyrtidae. Insects and Arachnids of Canada, (Part 13). 1985.

[33] Arnqvist G, Mårtensson T. Measurement error in geometric morphometrics: empirical strategies to assess and reduce its impact on measures of shape. Acta Zool Acad Sci Hungaricae. 1998;44:73–96.

[34] Sim LX, Zuha RM. *Chrysomya megacephala* (Fabricius, 1794) (Diptera: Calliphoridae) development by landmark-based geometric morphometrics of cephalopharyngeal skeleton: a preliminary assessment for forensic entomology application. Egypt J Forensic Sci. 2019;9:55.

[35] Smith K. A manual of forensic entomology. London (UK): British Museum (Natural History); 1986.

[36] Rognes K, Paterson H. *Chrysomya chloropyga* (Wiedemann, 1818) and *C. putoria* (Wiedemann, 1830) (Diptera: Calliphoridae) are two different species. African Entomol. 2005;13:49–70.

[37] Williams K, Villet M. Morphological identification of *Lucilia sericata*, *Lucilia cuprina* and their hybrids (Diptera, Calliphoridae). Zookeys. 2014;420:69–85. 10.3897/zookeys.420.7645 PMC4109482 25061373

[38] Frątczak K, Matuszewski S. Classification of forensically-relevant larvae according to instar in a closely related species of carrion beetles (Coleoptera: Silphidae: Silphinae). Forensic Sci Med Pathol. 2016;12:193–197. 27071758 10.1007/s12024-016-9774-0 PMC4859850

[39] Schawaller W. Faunistische und systematische Daten zur Silphiden-Fauna Sudafrikas (Coleoptera, Silphidae). Entomofauna. 1987;8:277–288. German.

[40] Von Lengerken H. Studien über die Lebenserscheinungen der Silphini (Coleopt.) xi – xiii. *Thanatophilus sinuatus* F., *rugosus* L. und *dispar* Hrbst. Zoomorphology. 1937;33:654–666. German.

[41] Anderson R. The Larva of *Thanatophilus trituberculatus* (Kirby) (Coleoptera: Silphidae). Coleopt. Bull. 1987;41:34.

[42] Villet MH, Richards CS, Midgley JM. Precision, bias and accuracy in development based estimates of PMI. Proc 7th Meet Eur Assoc Forensic Entomol. 2009.

[43] Midgley JM, Villet MH. Effect of the killing method on post-mortem change in length of larvae of *Thanatophilus micans* (Fabricius 1794) (Coleoptera: Silphidae) stored in 70% ethanol. Int J Legal Med. 2009;123:103–108. 18587595 10.1007/s00414-008-0260-4

[44] Villet MH. An inexpensive geometrical micrometer for measuring small, live insects quickly without harming them. Entomol Exper Applic. 2007;122:279–280.

[45] Bugelli V, Campobasso C, Pietro Verhoff MA, et al. Effects of different storage and measuring methods on larval length values for the blow flies (Diptera: Calliphoridae) *Lucilia sericata* and *Calliphora vicina*. Sci Justice. 2017;57:159–164. 28454623 10.1016/j.scijus.2016.10.008

[46] Mullin SK, Taylor PJ. The effects of parallax on geometric morphometric data. Comput Biol Med. 2002;32:455–464. 12356495 10.1016/s0010-4825(02)00037-9

[47] Day DM, Wallman JF. Width as an alternative measurement to length for post-mortem interval estimations using *Calliphora augur* (Diptera: Calliphoridae) larvae. Forensic Sci Int. 2006;159:158–167. 16188411 10.1016/j.forsciint.2005.07.009

[48] Adams Z, Hall M. Methods used for the killing and preservation of blowfly larvae, and their effect on post-mortem larval length. Forensic Sci Int. 2003;138:50–61. 14642719 10.1016/j.forsciint.2003.08.010

[49] Eliza P, Zuha RM. Preliminary assessment of cephalopharyngeal skeleton length and body length of *Hemipyrellia ligurriens* (Wiedemann) (Diptera: Calliphoridae) larvae as potential parameters to estimate minimum post mortem interval. Egypt J Forensic Sci. 2018;8:39.

[50] Bourne DR, Kyle CJ, LeBlanc HN, et al. Technical note: a rapid, non-invasive method for measuring live or preserved insect specimens using digital image analysis. Forensic Sci Int. 2019;1:140–145. 10.1016/j.fsisyn.2019.07.006 PMC7219176 32411966

[51] Grassberger M, Reiter C. Effect of temperature on *Lucilia sericata* (Diptera: Calliphoridae) development with special reference to the isomegalen- and isomorphen-diagram. Forensic Sci Int. 2001;120:32–36. 11457606 10.1016/s0379-0738(01)00413-3

[52] Richards CS, Paterson ID, Villet MH. Estimating the age of immature *Chrysomya albiceps* (Diptera: Calliphoridae), correcting for temperature and geographical latitude. Int J Legal Med. 2008;122:271–279. 17899152 10.1007/s00414-007-0201-7

[53] Grassberger M, Reiter C. Effect of temperature on development of the forensically important holarctic blow fly *Protophormia terraenovae* (Robineau-Desvoidy) (Diptera: Calliphoridae). Forensic Sci Int. 2002;128:177–182. 12175962 10.1016/s0379-0738(02)00199-8

[54] Minnich B, Lametschwandtner A. Lengths measurements in microvascular corrosion castings: two-dimensional versus three-dimensional morphometry. Scanning. 2000;22:173–177. 10888123 10.1002/sca.4950220305

[55] Tuxen S. The Protura: a revision of the species of the world with keys for determination. Paris (France): Hermann; 1964.

[56] Bunch AJ, Walters CJ, Coggins LG. Measurement error in fish lengths: evaluation and management implications. Fisheries. 2013;38:320–326.

